# Simultaneous Exposure to Heavy Metals among Residents in the Industrial Complex: Korean National Cohort Study

**DOI:** 10.3390/ijerph120605905

**Published:** 2015-05-27

**Authors:** Heejin Park, Kyoungho Lee, Chan-Seok Moon, Kyungsook Woo, Tack-Shin Kang, Eun-Kyung Chung, Bu-Soon Son

**Affiliations:** 1 Department of Environmental Health Science, Soonchunhyang University, 22 Soonchunhyang-ro, Asan-si, 336-745, Korea; E-Mails: hjpark83@sch.ac.kr (H.P.); wooks05@naver.com (K.W.); 2 Department of Occupational Epidemiology, Samsung Health Research Institute, Samsung Electronics, Suwon city 443-742, Korea; E-Mail: khlee3789@gmail.com; 3 Department of Industrial Health, Catholic University of Pusan, 57 Oryundae-ro, Geumjeong-gu, Busan, 609-817, Korea; E-Mail: csmoon@cup.ac.kr; 4 Department of Environmental Health, National Institute of Environment Research, 42 Hwangyeong-ro, Incheon, 404-170, Korea; E-Mail: lm8080@korea.kr; 5 School of Public Health, Seoul National University, 1 Gwanak-ro, Seoul, 151-015, Korea; E-Mail: ekchung88@naver.com

**Keywords:** heavy metal, industry complex, bio-monitoring, Cd, Pb, Hg

## Abstract

A survey was conducted to evaluate the multi-exposure level and correlation among toxic metal biomarkers (Cd, Pb, and Hg). A total of 592 individuals who participated in the survey were residents near an industrial complex in Gwangyang and Yeosu (exposed group) and of Hadong and Namhae (control group) in southern Korea from May 2007 to November 2010. The Gwangyang and Yeosu area exposed groups had slightly higher blood Pb (2.21 and 1.90 µg/dL), urinary Cd observed values (2.20 and 1.46 µg/L), urinary Cd with a urinary creatinine correction (1.43 and 1.25 µg/g Cr), and urinary Hg observed values (2.26 and 0.98 µg/L) in women participants than those in the Hadong and Namhae area (control group). Blood Pb (3.18 and 2.55 µg/dL), urinary Hg observed values (1.14 and 0.92 µg/L), and urinary Hg with a urinary creatinine correction (1.06 and 0.96 µg/L) for male participants were also slightly higher than those in the Hadong and Namhae area (control group). The correlation among urinary Cd, Hg and Pb concentrations in the blood was significant. We suggest that the exposed group of residents were simultaneously exposed to Pb, Cd, and Hg from contaminated ambient air originating from the iron manufacturing industrial complex.

## 1. Introduction

National and international environmental pollution and disease problems continue to rise, and the WHO reported that 25%–35% of diseases in developed countries are due to environmental factors [1]. The South Korean government has led the development of petrochemical, non-ferrous metal, shipbuilding, automotive industries, and urbanization since the 1960s, according to heavy chemical industry development policies. Thus, the type and amount of harmful substances entering the environment has increased rapidly, and the chances for exposure have increased [2,3,4]. Environmental policies are no longer sufficient to prevent pollution and protect the public, which deserves safe environmental quality [2,5]. The country’s leading industrial parks, container terminals, steel and thermal power plants, and other industries are arranged primarily in Gwangyang Bay, as a symbol of Korean’s industrial area [6]. The pollution generated due to increased industrial demand not only affects the surrounding areas of this industrial park but continues throughout society [7]. Health problems are emerging as an important issue in these areas with the increased use of various heavy metals [2,8,9]. The Environmental Protection Agency in the USA, the National Environmental Exposure Concentration [10], and the National Institute for Occupational Safety and Health (NIOSH) have assessed the impact of environmental pollutants through a variety of occupational disease systems. In particular, the Adult Blood Lead Epidemiology and Surveillance analyzes blood lead (Pb) levels. If blood Pb level is >25 µg/dL, it is directly reported to the main monitoring center, and they analyze and send information about the number of cases, people, age distribution, and concentration to the NIOSH who operates a reporting system [11]. Cadmium (Cd) accumulates and damages the liver, kidney, and reproductive organs, and also accumulates in the cardiovascular system [12]. As Cd exposure increases, the impact on the cardiovascular disease becomes more serious [13,14]. Pb is harmful to the nervous, renal, and endocrine systems, and low concentrations can elevate blood pressure and increase the risk of heart disease [15,16]. Mercury (Hg) accumulates in the body and damages the central nervous system as well as increases the prevalence of cardiovascular disease [17], coronary heart disease, and mortality [18]. However, the accumulation of heavy metals in the body is affected by age, ethnicity, and lifestyle and is also influenced by the intensity and duration of exposure. Environmental epidemiology studies are needed to evaluate the effects of the current level of environmental pollution in the Gwangyang industrial complex.

In this study, we continuously and systematically monitored and evaluated Pb in blood (B-Pb), Cd in urine as an observed value (U-Cd), Cd in urine as urinary creatinine correction (U-Cd cr), Hg in urine as an observed value (U-Hg), and Hg in urine as urinary creatinine correction (U-Hg cr) in residents of the Gwangyang and Yeosu industrial complex areas.

## 2. Methods

### 2.1. Geographic Features 

**Figure 1:**
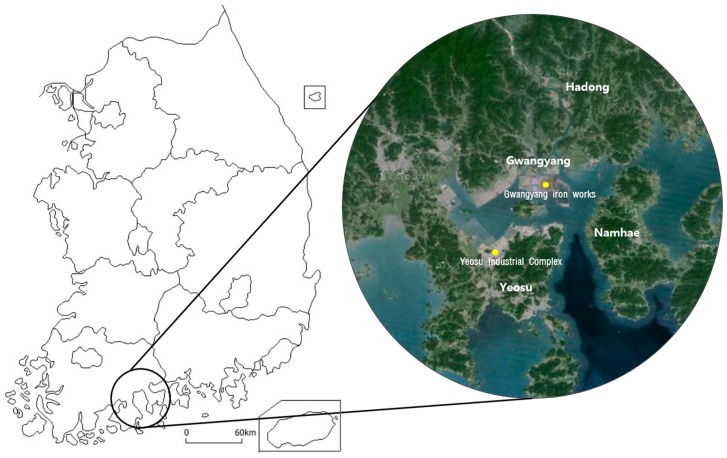
Map of the Gwangyang bay industrial complex in the Republic of Korea.

The east-west distance across Gwangyang Bay is 27 km, and it is 15 km wide from north to south. The Bay is surrounded by Gwangyang, Yeosu, Namhae, Hadong as a closed-coast; thus, pollutants spread out and become isolated in this area (Figure 1). The wind flows mainly south in summer and northeast in winter, which influences the damage caused by the air pollutants. In addition, water depth is low and the ocean tidal flow is slow; thus, water recovery from pollution is weak. Gwangyang Bay is adjacent to a residential area, and the pollutants emitted travel with the sea breeze toward a residential area.

### 2.2. Subjects and Areas

The participants in the exposed group were residents in the Gwangyang Bay area. Toxic metal (Pb, Cd, Hg) assessments were performed intensively from May 2007 to November 2010, in the Gwangyang and Yeosu National Industrial Complex around Gwangyang Steel works as representative industrial areas in South Korea. Gwangyang and Yeosu aimed the Residents who lived within 5 km with National industrial complex as the center. The Hadong and Namhae aimed the residents who lived over than 5 km with National industrial complex as the center. Response: We have conducted a multiple linear regression analysis, after adding to exposure and control areas. Residents of Hadong and Namhae areas were the control group with limited or no occupational and environmental exposure to Pb, Cd, or Hg. The subjects (n = 592) for the study were collected from those who agreed to participate (n = 2838). Subjects were excluded if they had an unclear address, no biological sample (blood and urine) data, or their and urinary creatinine concentration was out of the range of 0.5–3.0. This study was approved by the Institutional Review Board of Soonchunhyang University.

### 2.3. Sampling and Survey

Six ml of whole blood was collected in a Vacutainer (BD Science, Parsippany, NJ, USA) green capped tube containing heparin for the Pb measurements. The samples were carried to the laboratory at 4 °C and frozen at −20 °C. After thawing, we used a roller mixer to re-mix the samples before analysis. Urine was collected in a specimen cup, transferred to a 15 mL conical tube, and delivered refrigerated (4 °C) to the laboratory for the analysis of urinary Cd and Hg. The urine was frozen at −20 °C until analysis, which was performed within 24 h. The final heavy metal concentrations in the urine were revised to µg/g creatinine (cr). A survey of the enrolled subjects was performed by personnel who were trained sufficiently through interviews, and a standardized questionnaire derived from the Korea National Institute of Environmental Research was used. Information, lifestyle, demographic characteristics, dietary habits, diseases and drugs were included in this questionnaire survey.

### 2.4. Sample Analysis

The analysis of B-Pb and U-Cd were performed with a GF-AA instrument (SOLLAR M6, Thremo, Cambridge, England). The limits of detection (LODs) were 0.1 and 0.3 µg/dL, respectively. Hg was analyzed with the Milestone DMA-80 direct mercury analyzer (Shelton, CT, USA), and the LOD was 0.1 µg/dL.

Standard Reference Material was used for the internal quality control (National Institute of Standards and Technology) product for Pb in the blood. During the test, the analyzing was performed in Lot and almost all results were correct (90% of total items).

### 2.5. Statistical Analysis

We used Excel (Microsoft Corp. Redmond, WA, USA) for heavy metal data collection and the SPSS ver. 20.0 software package (SPSS Inc., Chicago, IL, USA) for the statistical analysis. The general characteristics of the study subjects and the geometric mean concentrations of heavy metals are shown as averages. We compared the average differences in the heavy metal concentrations by Student’s *t*-test. Pearson’s correlation coefficients were used to investigate the relationship among factors affecting blood and urinary heavy metal concentrations. For studying the factors, which effected on the concentration of the heavy metal, we carried out a multiple regression analysis. A *p* < 0.05 was considered significant.

## 3. Results

The 592 participant’s (median age, 58 years; 48% men and 52% women) characteristics and levels of Pb, Cd, and Hg are shown in Table 1. About 70% of the participants were nonsmokers. Mean body mass index was 23.7 kg/m^3^. The median B-Pb concentration of the 592 subjects was 2.55 µg/dL and 0.98 and 5.09 µg/dL were the 5th and 95th percentile values. Median U-Cd concentration was 1.77 µg/L (0.33 and 6.98 µg/L were the 5th and 95th percentiles). The U-Cd Cr was 1.32 µg/g Cr (0.67 and 2.26 µg/g Cr were the 5th and 95th percentiles). The U-Hg was 1.18 µg/L (0.21 and 3.13 µg/L were the 5th and 95th percentiles). The U-Hg Cr, 1.07 µg/g cr (0.57 and 1.75 µg/g cr were the 5th and 95th percentiles).

**Table 1:** Participant characteristics and levels of biomarkers of Pb, Cd and Hg.

Variables	No.	Percent	Median	(P5-P95) ^1^
Study group(exposed/control)	592	63/37		
Sex (male/female)	592	48/52		
Smoking (never/former/current)	592	70/6/24		
Alcohol use (yes/no)	592	58/42		
Education levels (Uneducated/elementary school/Middle and High school/Over college)	592	19/34/31/16		
Income per month (Korean won, KRW) (< 1,000,000 / 1,000,000–2,000,000 / 2,000,000–4,000,000 / ≥ 4,000,000)	592	55/19/19/8		
Age	592		58.0	(27.3–78.0)
Height	592		161.0	(146.1–175.0)
Weight	592		60.0	(45.0–80.5)
BMI	592		23.7	(18.8–28.8)
Blood-Pb (µg/dL)	592		2.55	(0.98–5.09)
Urinary-Cd (µg/L)	592		1.77	(0.33–6.98)
Urinary-Cd cr (µg/g cr)	592		1.32	(0.67–2.26)
Urinary-Hg (µg/L)	592		1.18	(0.21–3.13)
Urinary-Hg cr (µg/g cr)	592		1.07	(0.57–1.75)

**^1^** 5 percentile and 95 percentile.

Table 2 and Table 3 show a comparison between the heavy metal biomarkers in the exposed and control groups of women and men. The number of participants in the exposed and control groups were 186 and 106 women candidates and 185 and 115 man candidates, respectively. blood-Pb, urinary-Cd, urinary-Cd creatinine, and U-Hg were slightly higher in women of the exposed group than those in the control group. The geometric means were 2.21 µg/dL in the exposed group and 1.90 µg/dL in the control group (*p* < 0.01) for lood-Pb; 2.20 µg/L in the exposed group and 1.46 µg/L in the control group (*p* < 0.01) for urinary-Cd;1.43 µg/L in the exposed group and 1.25 µg/L in the control group (*p* < 0.01) in urinary-Cd creatitnine; and 1.23 µg/L in the exposed group and 0.98 µg/L in the control group (*p* < 0.05) for urinary-Hg. The geometric mean of urinary-Hg creatinine in women tended to be higher in the exposed group than that in the control group, but the difference was not significant. Blood-Pb, urinary-Hg and urinary-Hg cr were slightly higher in the exposed group of men than those in the control group. The geometric means were 3.18 µg/dL in the exposed group and 2.55 µg/dL in the control group (*p* < 0.01) for blood-Pb; 1.14 µg/L in the exposed group and 0.92 µg/L in the control group (*p* < 0.05) for urinary-Hg; and 1.06 µg/L in the exposed group and 0.96 µg/L in the control group (*p* < 0.05) for urinary-Hg creatinine. The geometric means of urinary-Cd and urinary-Cd creatinine in men of the exposed group tended to be higher than those in the control group, but the difference was not significant.

The correlation analysis results between the blood-Pb, urinary-Cd, urinary-Cd creatinine, urinary-Hg, and urinary-Hg creatinine, blood-Pb are shown in Table 4. All Pb, Cd and Hg biomarkers were correlated except for the correlations between urinary-Cd and urinary-Hg creatinine, and between urinary-Cd creatinine and urinary-Hg. The level of lead in the blood is the factors that effected on the gender and BMI from multiple regression analysis. The levels of Cd were the factors which effected significantly on gender, age, education, smoking status in adjusted urinary creatinine. The levels of Hg in the urine, the BMI and smoking were the effected factors (Table 5).

**Table 2:** Participant characteristics and levels of biomarkers of Pb, Cd and Hg.

Variables	Exposed				Control		
	GM	GSD	(P5–P95)^1^		GM	GSD	(P5–P95) ^1^
B-Pb (µg/dL)	2.69	1.55	(1.28–5.09)		2.51	1.60	(0.98–4.76)
U-Cd (µg/L)	1.76 **	2.21	(0.52–6.98)		1.22	2.22	(0.33–4.25)
U-Cd cr (µg/g cr)	1.76 **	2.21	(0.53–5.16)		1.22	2.22	(0.33–4.25)
U-Hg (µg/L)	1.22	2.08	(0.27–2.94)		1.09	2.55	(0.18–2.88)
U-Hg cr (µg/g cr)	1.14 *	2.12	(0.30–3.08)		1.09	2.49	(0.18–2.76)

**^1^** 5 percentile and 95 percentile; * *p* < 0.05, ** *p* < 0.01 by student’s *t*-test between exposed and control groups.

**Table 3:** Comparison between biomarkers of exposed and control group of women and man candidates.

Variables	Women								Man						
	Exposed (186) ^1^				Control (106) ^1^				Exposed (185) ^1^				Control (115) ^1^		
	GM	GSD	(P5–P95) ^2^		GM	GSD	(P5–P95) ^1^		GM	GSD	(P5–P95) ^1^		GM	GSD	(P5–P95) ^1^
B-Pb (µg/dL)	2.21 **	1.71	(0.76–4.14)		1.90	1.73	(0.72–4.07)		3.18 **	1.59	(1.39–5.93)		2.55	1.47	(1.44–4.94)
U-Cd (µg/L)	2.20 **	2.49	(0.47–8.44)		1.46	2.58	(0.27–7.20)		1.55	2.64	(0.28–6.11)		1.46	2.22	(0.27–4.38)
U-Cd cr (µg/g cr)	1.43 **	1.45	(0.76–2.52)		1.25	1.50	(0.58–2.15)		1.21	1.52	(0.59–2.16)		1.17	1.38	(0.68–1.78)
U-Hg (µg/L)	1.23 *	2.26	(0.32–3.87)		0.98	2.31	(0.24–3.16)		1.14 *	2.05	(0.30–3.18)		0.92	2.08	(0.31–2.80)
U-Hg cr (µg/g cr)	1.12	1.40	(0.60–1.73)		1.06	1.42	(0.58–1.75)		1.06 *	1.38	(0.63–1.76)		0.96	1.40	(0.61–1.81)

**^1^** Number of participants; **^2^** 5 Percentile and 95 percentile; * *p* < 0.05, ** *p* < 0.01 by student’s *t*-test between exposed and control groups.

**Table 4:** Pearson correlation coefficient between biomarkers of Pb, Cd, Hg (No.=592).

Variables	U-Cd	U-Hg	U-Cd cr	U-Hg cr
B-Pb	0.102 *	0.182 **	0.091 *	0.170 **
U-Cd		0.224 **	0.857 **	0.056
U-Hg			0.025	0.838 **
U-Cd cr				0.170 **

* *p* < 0.05, ** *p* < 0.01.

**Table 5:** Participant characteristics and levels of biomarkers of Pb, Cd and Hg.

Variables	Factor	Β		Standard error	*p*-Value	R^2^
B-Pb	Constant	1.174		0.230	0.000	0.147
	Sex	−0.312		0.040	0.000	
	Age	−0.003		0.002	0.058	
	BMI	0.014		0.005	0.008	
	Area	−0.069		0.036	0.057	
	Education level	−0.023		0.017	0.172	
	Smoking	0.032		0.024	0.190	
U-Cd	Constant	−0.858		0.376	0.023	0.319
	Sex	0.356		0.065	0.000	
	Age	0.019		0.003	0.000	
	BMI	0.013		0.008	0.111	
	Area	0.264		0.059	0.000	
	Education level	−0.127		0.028	0.000	
	Smoking	0.109		0.040	0.006	
U-Cd cr	Constant	−0.856		0.376	0.023	0.320
	Sex	0.356		0.065	0.000	
	Age	0.019		0.003	0.000	
	BMI	0.013		0.008	0.110	
	Area	0.265		0.059	0.000	
	Education level	0.127		0.028	0.000	
	Smoking	0.109		0.040	0.006	
U-Hg	Constant	0.149		0.486	0.759	0.034
	Sex	0.013		0.084	0.874	
	Age	−0.006		0.004	0.082	
	BMI	0.028		0.011	0.008	
	Area	−0.121		0.076	0.111	
	Education level	−0.030		0.036	0.407	
	Smoking	−0.149		0.051	0.004	
U-Hg cr	Constant	−0.104		0.475	0.827	0.056
	Sex	0.158		0.082	0.054	
	Age	−0.005		0.004	0.196	
	BMI	0.029		0.010	0.006	
	Area	−0.181		0.074	0.015	
	Education level	−0.043		0.035	0.228	
	Smoking	−0.132		0.050	0.009	

## 4. Discussion

This study was the first national cohort study performed with residents who live in South Korea’s industrial complex area. We investigated heavy metal concentrations in biological samples among people who live in the Gwangyang and Yeosu industrial complex area.

The average B-Pb concentration was 2.21 µg/dL in women and 3.18 µg/dL in men of the exposed group, which was higher than those values in the control groups of men and women (1.90 µg/dL in women and 2.55 µg/dL in men). The average (control) Korean B-Pb concentration is 1.91 µg/dL [19], whereas it is 1.12 µg/dL the USA [11] and 1.34 µg/dL in Canada [20]. However, the B-Pb levels in this study were lower than those reported in Germany (3.07 µg/dL) [21] and Brazil (2.37 µg/dL) [22]. In this study, males (2.55 µg/dL) had higher B-Pb levels than those of females (1.90 µg/dL), which was similar to previous studies [21,22,23]. Males had higher B-Pb levels than those of females in German human biomonitoring commission research [24]. As age increases, blood concentrations of heavy metals increase, particularly in those between 40–59 years old, who have recorded the highest concentration of 2.56 µg/dL [2,25]. The age difference reflects the difference between the amount of activity and the environment. It is generally reported that older people’s B-Pb concentrations are higher, and that the rapid increase in B-Pb concentration is caused by postmenopausal women’s bone loss [25].

Women in the exposed group had significantly higher U-Cd (2.20 µg/L) and U-Cd cr (1.43 µg/g) than those in the control group (1.46 µg/L for U-Cd and 1.25 µg/g for U-Cd cr) (*p* < 0.01). The U-Cd levels in previous reports of a Korean (control) survey showed 1.23 µg/L for U-Cd and 1.56 µg/g for U-Cd cr [26]. The geometric mean U-Cd levels in other locations include 1.40 µg/g cr for U-Cd cr in Bangkok [27], 1.51 µg/g cr for U-Cd cr in Kuala Lumpur [28], 1.21 µg/g cr for U-Cd cr in Manila [29], and 1.59 µg/g cr for U-Cd cr in a Taiwan survey [30]. Higher U-Cd was observed in a Japanese study than the values observed in our study [31]. These reports show comparable values to our control group. However, the geometric mean U-Cd cr value in a Japanese study was higher (4.39 µg/g cr) than the results of our study (1.43 µg/g cr). Cooked rice as a main energy source is a strong determinant of variations in dietary Cd intake [31,32]. According to the US NHANES survey, the potential exposure risks for Cd in urine are occupational and smoking [11].

Because many factories exist in the industrial complex, the residents could be affected by Hg emissions into the atmosphere. Geometric means that could influence residents of the exposed group were 1.23 µg/L and 1.14 µg/L in U-Hg of women and man candidate. It was slightly higher (*p* < 0.05) than the concentration of control group (0.98 and 0.92 µg/L in U-Cd). However, in case of U-Hg, exposed group was significantly higher (1.12 and 1.06 µg/g cr in woman and man candidates) than control group (1.06 and 0.96 in woman and men) in men (*p* < 0.05), but not in women (*p* > 0.05). The results of the control group in our study were higher than those found in Germany (0.34 µg/g cr) [21], the United States (0.46 µg/g cr) [11], and Canada (0.40 µg/g cr) [20]. People living in the Gwangyang and Yeosu industrial complex could be affected by exposure to Hg, compared to the people living in area that is not influenced by Hg.

Pearson’s correlation coefficients were significant for almost all variables except for the correlation between U-Cd and U-Hg cr, and between U-Cd cr and U-Hg. We suppose that these three toxic metals are emitted simultaneously, so that a higher Pb exposure results in higher Cd and Hg exposure. Participants in the exposed group were slightly affected by ambient air exposure, although the main exposure source is from intake of food especially seafood containing these three metals [33]. More study is necessary regarding the exposure routes from food sources and contaminant ambient air.

## 5. Conclusions

We suggest that the exposed group of residents were simultaneously exposed to Pb, Cd, and Hg from contaminated ambient air originating from the iron manufacturing industrial complex. Residents who live in the Gwangyang and Yeosu areas should be systematically monitored and continuously evaluated for health effects. We need to research the health damage to residents in industrial areas to provide basic data for expanding and establishing a center for environmental pollution control measures.
